# Lower limb peripersonal space and the desire to amputate a leg

**DOI:** 10.1007/s00426-020-01316-1

**Published:** 2020-03-20

**Authors:** Kayla D. Stone, Clara A. E. Kornblad, Manja M. Engel, H. Chris Dijkerman, Rianne M. Blom, Anouk Keizer

**Affiliations:** 1 Department of Experimental Psychology, Helmholtz Institute, Utrecht University, Heidelberglaan 1, 3584 CS Utrecht, The Netherlands; 2 Department of Psychiatry, Amsterdam UMC, Vrije Universiteit Amsterdam, Amsterdam, The Netherlands

## Abstract

Body integrity identity disorder (BIID) is a rare condition defined by a persistent desire to amputate or paralyze a healthy limb (usually one or both of the legs). This desire arises from experiencing a mismatch between the internal body model and the actual physical/functional boundaries of the body. People with BIID show an abnormal physiological response to stimuli approaching the affected (unwanted) but not the unaffected leg, which might suggest a retracted peripersonal space (PPS: a multisensory integration zone near the body) around the unwanted limb. Thus, using a visuo-tactile interaction task, we examined leg PPS in a group of healthy men and three men with BIID who desired unilateral leg amputation. PPS size (~ 70 cm) around the unwanted BIID legs did not differ from that of healthy controls. Although the leg feels foreign in BIID, it still seems to maintain a PPS, presumably to protect it and facilitate interactions within the surrounding environment.

## Introduction

Approximately 30 years ago, single-cell recordings from the brains of non-human primates showed that there are special neurons in the frontal and parietal areas that respond not only to the presence of an object touching the body (e.g. a ball in contact with a body part) but also to the presence of the object near the body (e.g. a ball looming towards that same body part; Fogassi et al., 1996; Graziano, Yap, & Gross, 1994; Graziano, Hu, & Gross, 1997). These (multisensory) neurons have tactile receptive fields with overlapping visual/auditory receptive fields that extend into space around the body. The product of this neuronal activity is usually described as peripersonal space (PPS; Rizzolatti, Scandolara, Matelli, & Gentilucci, 1981). For example, if one is talking to a friend and a ball rolls towards her foot, she might retract her foot or shift her body so that the ball continues to roll by, not allowing it to intersect with her body and interrupt the conversation. Alternatively, she could choose to interact with the ball, likely kicking it away from the body towards the location it originated. In either case, PPS can be thought of as a probabilistic action space (Bufacchi & Iannetti, 2018), predicting the probability of contact with an object and therefore preparing the action that follows. In other words, PPS is often defined as the zone surrounding the body in which multisensory integration of sensory stimuli (e.g. vision of ball plus prediction of touch) readily transpires and thus these actions occur. PPS functions to defend the body (e.g. by quickly retracting the body part) but also to allow for interaction with the objects around the body (e.g. by kicking the ball), serving as an important interface between the self and the environment (Brozzoli et al., 2012; Serino et al., 2016).

While single-cell recordings looking at PPS have not been conducted in humans, neuroimaging and behavioral studies in healthy people and in patients suggest that we have a similar network which governs the interactions around the body (Bremmer et al., 2001; Canzoneri, Magosso, & Serino, 2012; Huang et al., 2012; Làdavas, di Pellegrino, Farnè, & Zeloni, 1998; Làdavas, Pavani, & Farnè, 2001). As in non-human primates, studies in humans reveal that PPS is body part centered, insofar that the distance from the body in which integration of tactile stimuli with looming visual/auditory stimuli occurs (i.e. the boundary of PPS) depends on body part. That is, PPS boundaries for the head (~ 60 cm), hands (~ 30–50 cm), trunk (~ 65 cm), and lower limbs (~ 75 cm) all reveal unique patterns of multisensory integration (Kandula, van der Stoep, Hofman, & Dijketman, 2017; Serino et al., 2016; Stone, Kandula, Keizer, & Dijkerman, 2018b). These boundaries are usually discerned by asking participants to respond to a tactile stimulus, often a vibration, on one of the body parts while a task-irrelevant visual (visuo-tactile interaction task) or auditory (audio-tactile interaction task) stimulus approaches that body part. Responses time and sensitivity to tactile stimuli are contingent upon the proximity of the visual or auditory stimulus to the body, insofar that tactile reaction times (and tactile detection; Làdavas, di Pellegrino, Farnè, & Zeloni, 1998; Làdavas, Pavani, & Farnè, 2001; Salomon, Noel, & Łukowska, 2017) are enhanced when the looming stimulus enters PPS. Furthermore, there is some recent evidence to suggest that PPS is not linked to the physical body per se, but to the experienced sense of self. Noel, Pfeiffer, Blanke, and Serino (2015b) induced a full-body illusion in participants by stroking their backs while they viewed their bodies (through a head-mounted display) at a location 2 m ahead. PPS was measured using an audio-tactile interaction task, by asking participant to make speeded responses to tactile stimuli on the chest while a task-irrelevant auditory stimulus loomed towards the body. They found that PPS boundaries shifted towards the virtual body, where participants felt they were located, demonstrating that PPS is not bounded to the physical body but to the experienced self*.*

People with body integrity identity disorder (or BIID) experience a mismatch between the physical body and their experienced self (Blom, Hennekam, & Denys, 2012). These individuals desire amputation or paralysis of a perfectly healthy body part, usually one or both legs. BIID is a non-psychotic condition that manifests before adolescence and in the absence of any apparent brain damage (Blom, Hennekam, & Denys, 2012; Brugger, Lenggenhager, & Giummarra, 2013; First & Fisher, 2012; van Dijk et al., 2013). The neural networks implicated in constructing a coherent representation of the body, especially the legs, seem to be altered in BIID compared to controls (Blom et al., 2016b; Hänggi et al., 2017; Hilti et al., 2013; McGeoch et al., 2011; Oddo-Sommerfeld et al., 2018; van Dijk et al., 2013). Furthermore, those who desire amputation (i.e. the amputation variant, also known as xenomelia; McGeoch et al., 2011), experience a sense of disownership over the affected limb(s), insofar that it does not belong to the body, and should be removed. Those with the paralysis variant (i.e. those who desire paralysis), however, are seemingly unbothered by the presence of legs but report instead that legs simply should not function and/or be felt (Giummarra et al., 2012). It has been suggested that while sensory input is normal in BIID (e.g. like the feeling of touch on the leg), it cannot integrate with a higher-order representation of the body to provide a sense of completeness in one’s body, leading to discomfort and even disownership (Hänggi et al., 2017; Ramachandran, Brang, McGeoch, & Rosar, 2009; Romano, Sedda, Brugger, & Bottini, 2015). Instead, people with BIID report feeling ‘overcomplete’ in the body, and that by structurally (through amputation) and/or functionally (through paralysis) modifying the legs would, counterintuitively, provide a feeling of completeness. Indeed, once an amputation is achieved, BIID is seemingly cured (Blom, Guglielmi, & Denys, 2016a; Noll & Kasten, 2014). In line with this, people with BIID report that their internal identity is that of an amputee or a paralyzed individual (First & Fisher, 2012).

If the limb is not properly inscribed into the body representation (Romano, Sedda, Brugger, & Bottini, 2015), one might wonder whether PPS boundaries around the affected limb are compromised in BIID. At least one piece of evidence speaks to this query. In one study, an experimenter approached the affected and unaffected legs of people with amputation variant BIID with a pin and/or cotton swab (Romano, Sedda, Brugger, & Bottini, 2015). Approaching the unaffected leg led to typical anticipatory skin conductance responses, i.e. an increase in skin conductance as the stimulus got closer. Approaching the affected leg, however, revealed negligible skin conductance responses (SCR), even though the approaching stimulus was in plain sight. What is more, once the stimulus made contact with the affected leg, there was an exaggerated (when compared to the unaffected leg) SCR, as if the brain did not anticipate the looming stimuli. The authors therefore suggested that the affected leg fails to be inscribed into the higher-order body representation and that “such an under-representation might induce a scarce attention for any signal coming from the environment directed to the limb felt as outside from the body representation” (p. 146, Romano, Sedda, Brugger, & Bottini, 2015). We propose that this failure to anticipate contact with the affected leg might also be a reflection of a disturbed, possibly diminished, PPS around the affected leg. Furthermore, individuals with BIID desire to decrease (or completely abolish) the function of their affected limb(s). While no study, to our knowledge, has explored whether BIID is rooted in problems related to action, it is feasible that it could be. As PPS is usually characterized as an “action-space” around the body, exploring it in people with BIID could shed light on this possibility.

Therefore, the aim of the current study was to investigate the shape and size of leg PPS in healthy individuals and in individuals with BIID. Mimicking Stone, Bullock, Keizer, and Dijkerman (2018a) and Stone, Kandula, Keizer, and Dijkerman (2018b), we used a multisensory visuo-tactile interaction paradigm around the feet to assess PPS. Participants were asked to respond to tactile stimuli on their toes, while a task-irrelevant visual stimulus approached the left or right foot. We hypothesized that participants with BIID would have smaller PPSs around the affected (i.e. desired to-be-removed) legs in comparison to the same legs of controls and their unaffected leg. Understanding PPS around the legs in BIID might provide insight into the mechanisms underlying it.

## Methods

### Materials and procedures

#### Participants

##### BIID participants

Three males with a unilateral lower limb amputation desire took part in the study. They are described in Table 1. These participants were recruited through online support group forums (https://groups.yahoo.com/neo/groups/fighting-it/ and https://forum.biid.ch/) and via collaboration with another BIID researcher (Blom, van der Wal, Vulink, & Denys, 2017). Each BIID participant was called by a psychiatrist prior to their participation to confirm the desire to change the body arose from having BIID and was not a product of another psychiatric condition. The criteria from First and Fisher (2012) was used as a guideline to confirm BIID in the participant. Participants were asked about the history of the BIID, the presence of psychiatric illnesses, and whether they had normal tactile sensitivity and vision. All contacted participants were eligible for participation. For a more thorough assessment of the individual’s psychiatric profile, a trained neuropsychologist administered the Structured Clinical Interview for the DSM-5 axis I and axis II (SCID-5) disorders on the day of testing in Utrecht. Psychiatric profiles were overall normal for all three participants. Following are descriptions of each BIID participant.

**Table 1:** Characteristics of BIID sample

Participant	Sex	Age	Desire (lower limbs only)	Age of BIID onset
1-LA	M	51	Amputation: left	6
2-LA	M	42	Amputation: left	7
3-RA	M	42	Amputation: right	6

Under the ‘Participant’ column, ‘L’ represents left leg and ‘R’ represents right leg while ‘A’ represents amputation desire

1-LA was a 51-year-old man with a desire for a left leg amputation, approximately 10 cm above his knee. He stated that he had this desire since he was 6 years old. He reported that the realization of this feeling was triggered by putting his bent left leg in his pants and looking at the ‘empty part’ (i.e. the simulation of an amputation). He was not currently taking any medication at the time of testing. He had obtained some form of higher education degree.

2-LA was a 42-year-old man with a desire for left leg amputation, approximately 10 cm above his knee. He had this desire since age 7 but could not recall a trigger at the time the BIID started. The only medication he was taking at the time of testing was insulin as he had diabetes mellitus. He had obtained a university degree.

3-RA was a 42-year-old man with a desire for right leg amputation, approximately 10 cm above his knee. He had this desire since around age 6 and also could not recall a trigger at the time the BIID started. He was taking an antidepressant at the time of testing, for which he had been on for approximately 1 year. His highest level of education obtained was secondary school.

##### Control participants

Sixteen male participants [average age 40.1 years (14.7 SD)] took part in the experiment. Highest level of education completed was as follows: university (*n* = 4), higher education (*n* = 5), secondary school (*n* = 3). Education level was missing for the remaining four participants. All participants had normal or corrected-to-normal vision and reported normal tactile sensitivity. Participants reported no current psychiatric illnesses, and this was corroborated by our screenings with the modified MINI screen (Sheehan et al., 1998) and a SCID-5 questionnaire for personality disorders (First, 2015). However, the screenings were missing for five participants as they were tested at an earlier date prior to implementing those screenings. Participants were recruited via online study participant websites, Utrecht University’s intranet, and word of mouth.

#### Questionnaires

##### General demographic

All participants completed a general questionnaire about their demographics and medical history. To gain more insight into our BIID sample, these individuals completed a more elaborate version that all included questions about their BIID [modified version of BIID Phenomenology Questionnaire by Blomet al., (2012)]. These results were used to describe our participant sample.

##### 12-item Zurich Xenomelia Scale

This has been described by us in the supplementary material of Stone, Bullock, Keizer, and Dijkerman (2018a) and Stone, Kandula, Keizer, and Dijkerman (2018b). The 12-item Zurich Xenomelia Scale (ZXS) (Aoyama et al., 2012) consists of three subscales regarding (1) the strength of the participant’s amputation (or paralysis) desire, (2) the participant’s erotic attraction to amputees/being an amputee, and (3) the extent to which the participant engages in pretending behaviors (i.e. simulated the bodily state of being amputated or paralyzed). Participants rated their agreement with each statement from 1 (strongly agree) to 6 (strongly disagree).

#### Visuo-tactile interaction task

Methods were similar to Stone, Bullock, Keizer, and Dijkerman (2018a) and Stone, Kandula, Keizer, and Dijkerman (2018b) and are described in more detail there. The task was run using a custom MATLAB (version R2015b) script. The visual stimulus consisted of a 5 cm (in diameter) red opaque circle projected onto a black cloth on the floor, which loomed towards the participant’s toe at 33.5 cm/second. Visual stimuli were presented via a projector (model: SANYO ProXtraX, positioned at 110 cm H) which reflected onto an adjacent mirror (165 cm away, dimensions: 100 cm L × 150 cm W, attached to 80 cm H legs), angled toward the floor at 40°, where the stimuli were seen. The projection consisted of a black rectangle (60 cm W × 143.5 cm L) with a red opaque circle that traveled along the length of the rectangle towards the participants’ toe. An 8 mm vibrotactile motor (Precision Microdrives, model: 308-00) delivered a vibration (200 Hz, for 100 ms) to the participant’s left or right big toe on 50% of trials and only once per trial. To reduce tactile expectation effects, which might lie outside of the PPS system (Kandula, van der Stoep, Hofman, & Dijkerman, 2017), the remaining 50% of trial were catch trials (no tactile stimulus (Kandula, Hofman, & Dijkerman, 2015; Stone, Kandula, Keizer, & Dijkerman, 2018b). Participants were seated comfortably at a chair, feet flat on the floor (30 cm apart) on the short edge of the projection site, with their right hand resting on a motor box situated on the participants’ right side. They were instructed to press a button on the motor box whenever they felt a vibration on the toe.

**Fig. 1:**
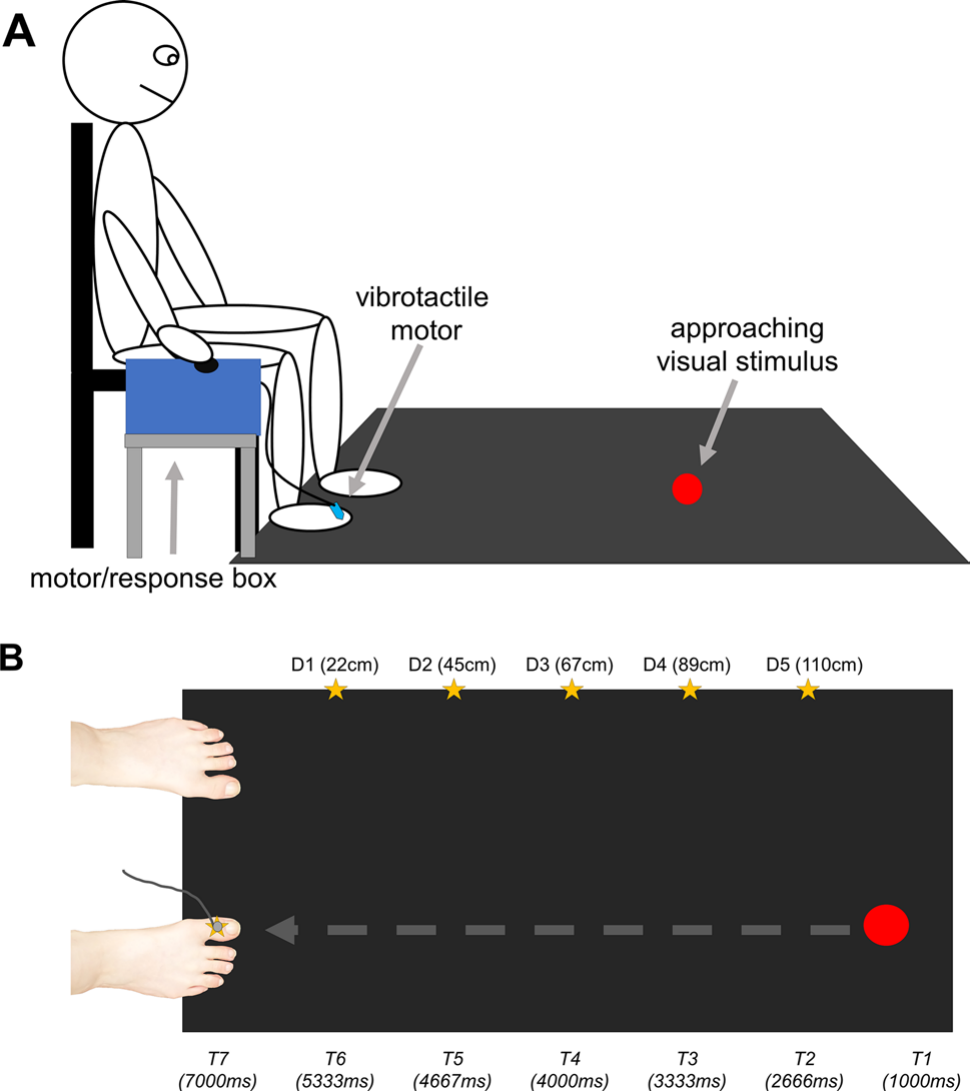
**a** Pictorial example of visuo-tactile interaction task for the right foot. **b** Bird’s eye view of task setup. The horizontal grey-dashed arrow indicates the visual stimulus (red circle) trajectory. The stars represent possible points (and thereby distances from the toe) in which a tactile stimulation to the toe could be given. *D* represents visual stimulus distance from the toe and *T* represents time in milliseconds from the start of the trial. *D*s and *T*s have been rounded to the nearest whole number

The vibration could occur at one of seven time points (*T*) after the trial started (*T*1: 1000 ms, *T*2: 2666 ms, *T*3: 3333 ms, *T*4: 4000 ms, *T*5: 4667, *T*6: 5333 ms, *T*7: 7000 ms). During time points 2–6, the visual stimulus was onscreen, located approximately at the following distances (*D*) from the participants’ foot: *T*6 (*D*1) = 22.23 cm, *T*5 (*D*2) = 44.46 cm, *T*4 (*D*3) = 66.69 cm, *T*3 (*D*4) = 88.92 cm, *T*2 (*D*5) = 111.15 cm). So as time passed, the distance between the participants’ toes and the visual stimulus became shorter. *T*1 and *T*7 (occurring at the start and end of the trial, respectively) were not accompanied by a visual stimulus and were thus coded as unimodal trials, to calculate a baseline reaction time for tactile stimuli. Tactile stimuli time (i.e. *T*1–*T*7) was randomized within and between each participant. There was a total of 84 tactile trials and 84 catch trials, interspersed with one another. Participants completed the task twice, once per foot. To make sure participants could feel the vibrations, we administered three individual vibrations (plus two catch trials: no vibration) to participants’ big toes prior to experiment. Participants had to state if they felt it and its intensity. All included participants indicated that they felt all applied vibrations and did not report feeling a stimulus on the catch trials. Moreover, to familiarize participants with the task, there was also a short practice block of ten trials (not included in the analysis) prior to task initiation. See Fig. 1 for visualization of setup and procedure.

#### Data analysis

Data were analyzed using MATLAB (version R2015b) and SPSS (version 23). Mean reaction times per tactile stimulus were obtained. Tactile reaction times beyond two standard deviations of the participants’ mean reaction per visual location, or missed trials, were removed from analysis (7.6% for controls, 9.3% for BIID).

##### Distance analysis

A 5 (distance) × 2 (leg) repeated-measures ANOVA was conducted on the mean reaction times for control participants to examine if they responded faster to the tactile stimulus as the stimulus got closer to the foot.

##### Curve fitting

To explore the relationship between the visual stimulus and tactile reaction times, mean reaction times per distance and per participant (for bimodal visuo-tactile trials only) were fitted to and compared between two mathematical models: linear and sigmoidal (Canzoneri, Magosso, & Serino, 2012; Kandula, Hofman, & Dijkerman, 2015; Serino et al., 2016; Stone, Kandula, Keizer, & Dijkerman, 2018b). If the data are better explained by a sigmoid [in terms of *R*^2^ and root mean squared error (RMSE)], then one can take the central point (‘a’ in the following equation) as the PPS boundary (in terms of distance, i.e. the point in space where reaction times were suddenly facilitated by the location of the visual stimulus). If the data are better explained by a linear model, then that suggests that the increase in reaction times does not suddenly increase as some point in space, but rather increases steadily as a function of visual stimulus, such that a boundary between peri- and extra-personal space cannot necessarily be discerned (e.g. Canzoneri, Magosso, & Serino, 2012).

The equation for the sigmoidal function was:

$$ y\left( x \right) = y_{\min } + \left( {\frac{{\left( {y_{\max } - y_{\min } } \right)}}{{\left( {1 + e^{{\left( {\left( {a - x} \right) \times b} \right)}} } \right)}}} \right)$$

where *y* represents the reaction time to the tactile stimulus (in milliseconds), *b* represents the slope, *x* represents the location of the visual stimulus, and *a* represents the central point of the sigmoid.

The equation for the linear function was:

$$y\left( x \right) = a \times x + b$$

where *y* represents the reaction time to the tactile stimulus (in ms), *a* represents the slope, *x* represents the location of the visual stimulus, and *b* represents the *y*-intercept.

##### Comparison to BIID participants

Goodness-of-fit values (RMSE and *R*^2^) were calculated for each BIID participant per leg per mathematical model using the mean reaction times. A multiple single-case approach was used to compare the fits between the BIID participants (individually) and the control subjects. Crawford–Garthwaite Bayesian single-case *t* tests (Crawford & Garthwaite, 2007) in *R* using the psycho package (Makowski, 2018) were used to compare between BIID and controls. Bayesian tests on the difference between the case’s standardized scores (BSTDs) were used to examine whether the PPS boundary for the affected and unaffected leg in the BIID participants differed (using the progam: DissocsBayes_ES_CP from Crawford & Garthwaite, 2007; Crawford, Garthwaite, & Porter, 2010).

Where relevant, for group-level comparisons, Cohen’s *d* and partial eta squared (*η*2) are used to show effect sizes. Greenhouse–Geisser correction is used when the assumption of sphericity is violated. For single-case comparisons, effect size (*Z*) for the difference between case and controls (CC) is represented as *Z*-CC or *Z*-*D*CC for difference (*D*) between case and controls between right and left legs.

## Results

### 12-item Zurich Xenomelia Scale (BIID participants only)

Scores for each subscale of the Zurich Xenomelia Scale per participant are reported in Table 2. Overall, these scores are in line with previous studies using this scale to describe their BIID sample (e.g. Aoyama et al., 2012; Hänggi, Bellwald, & Brugger, 2016; Hilti et al., 2013).

**Table 2:** 12-item Zurich Xenomelia Scale scores per BIID participant

Subject	Desire (lower limbs)	Pure amputation	Erotic attraction	Pretending behaviors
1-LA	Amputation: left	5.5	1.5	4.5
2-LA	Amputation: left	6	5	5.5
3-RA	Amputation: right	5.5	4.25	4.75
	Average	5.6	3.5	4.9

The total score is the average of all scores. Scores could range from 1 to 6, with higher scores indicating stronger BIID symptomatology

### Visuo-tactile interaction task

#### Control participants

##### Distance analysis

A 5 (distance) × 2 (leg) repeated-measures ANOVA was conducted on the mean reaction times to see if participants responded faster to the tactile stimulus as the stimulus got closer to the foot. There was a main effect of distance (*F*(2.33,44.94) = 4.9, *p* = 0.002, *η*2 = 0.24). Bonferroni-corrected pairwise comparisons revealed that *D*5 significantly differed from *D*3 (*p* = 0.017), *D*2 (*p* = 0.006), and *D*1 (*p* = 0.004), but not from *D*4 (*p* = 0.41). *D*4 did not differ from *D*1, *D*2, or *D*3 (*p* > 0.9 for all). *D*1, *D*2, and *D*3 also did not differ from one another (*p* = 1.0). There was a main effect of side (*F*(1,15) = 4.4, *p* = 0.053, *η*2 = 0.22), indicating that participants were significantly faster at responding to tactile stimuli on the right foot (483 ms ± 85 SD) than the left foot (502 ms ± 87 SD). However, there was no interaction between distance and side (*F*(4, 60) = 0.6, *p* = 0.61, *η*2 = 0.04).

##### Curve-fitting analysis

**Fig. 2:**
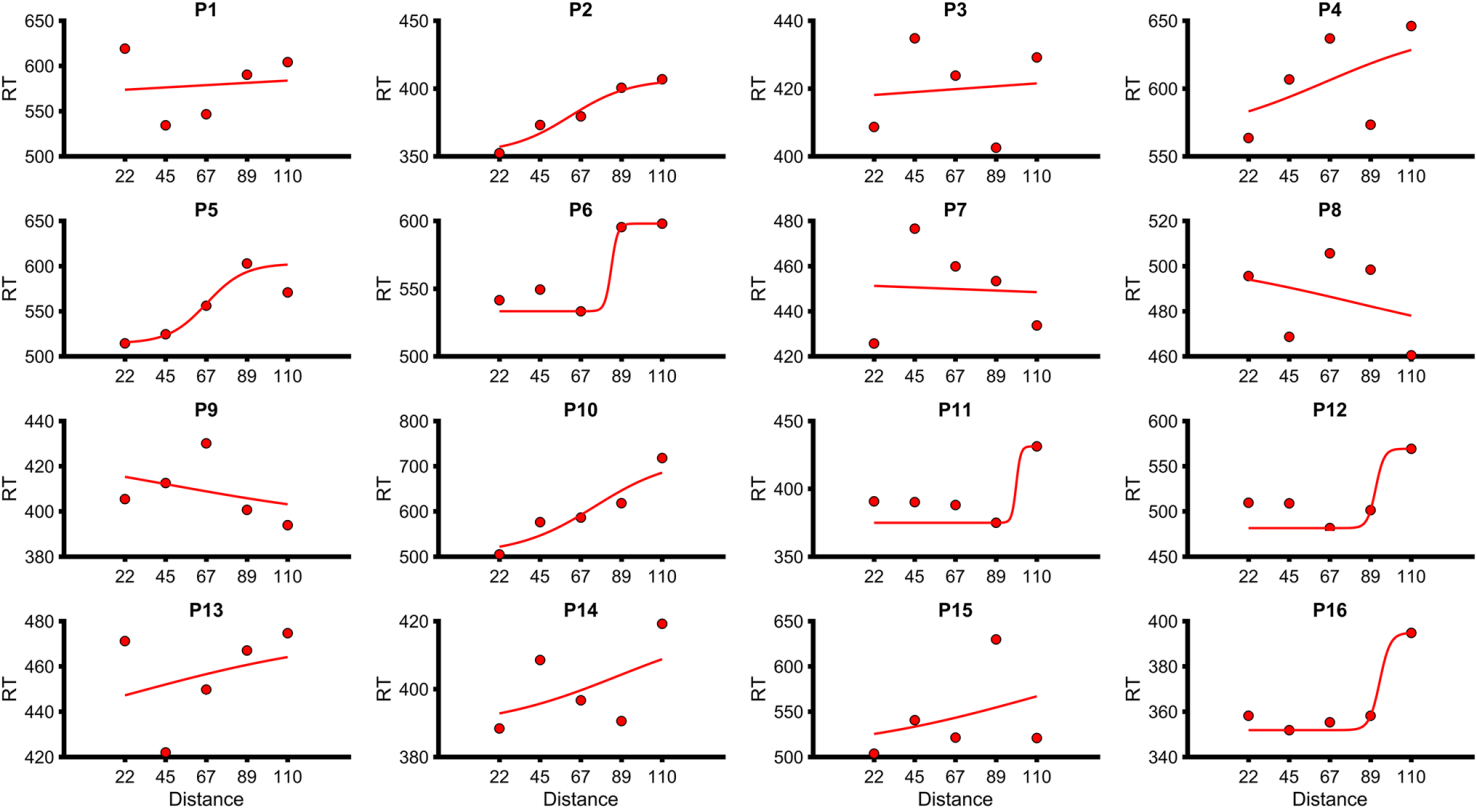
Mean reaction times (represented as red opaque circles) and sigmoidal fits (red lines) for the right leg of individual control participants (represented a *P* for participant followed by participant number). The *y*-axis represents the reaction time (RT) in milliseconds and the *x*-axis represents the distance from the foot (in centimeters) that the visual stimulus was located when the tactile stimulus was administered

*Right leg* Shapiro–Wilk tests revealed that the *R*^2^ values for the linear (*W* = 0.87,* p* = 0.035) and the sigmoidal (*W* = 0.84,* p* = 0.01) fits were not normally distributed. Thus, Wilcoxon signed-rank tests were conducted. There was no difference between linear (median = 0.26, IQR = 0.13–0.54) and sigmoidal (median = 0.28, IQR = 0.11–0.81) fits based on the *R*^2^ (*W* = 74, *p* = 0.7). Paired samples *t* tests revealed that RMSEs (in ms) for the sigmoidal fits (22.0 ± 13.3 SD) were not different from the linear fits (23.9 ± 12.0 SD; *t*(15) = − 1.9, *p* = 0.1, *d* = − 0.4). Since there was no difference between the two fits, we took a pragmatic approach and chose a sigmoid fit. Therefore, the central point of the sigmoid was taken as the PPS boundary (68.8 cm ± 26.1). See Fig. 2 for visualization of individual sigmoid fits.

**Fig. 3:**
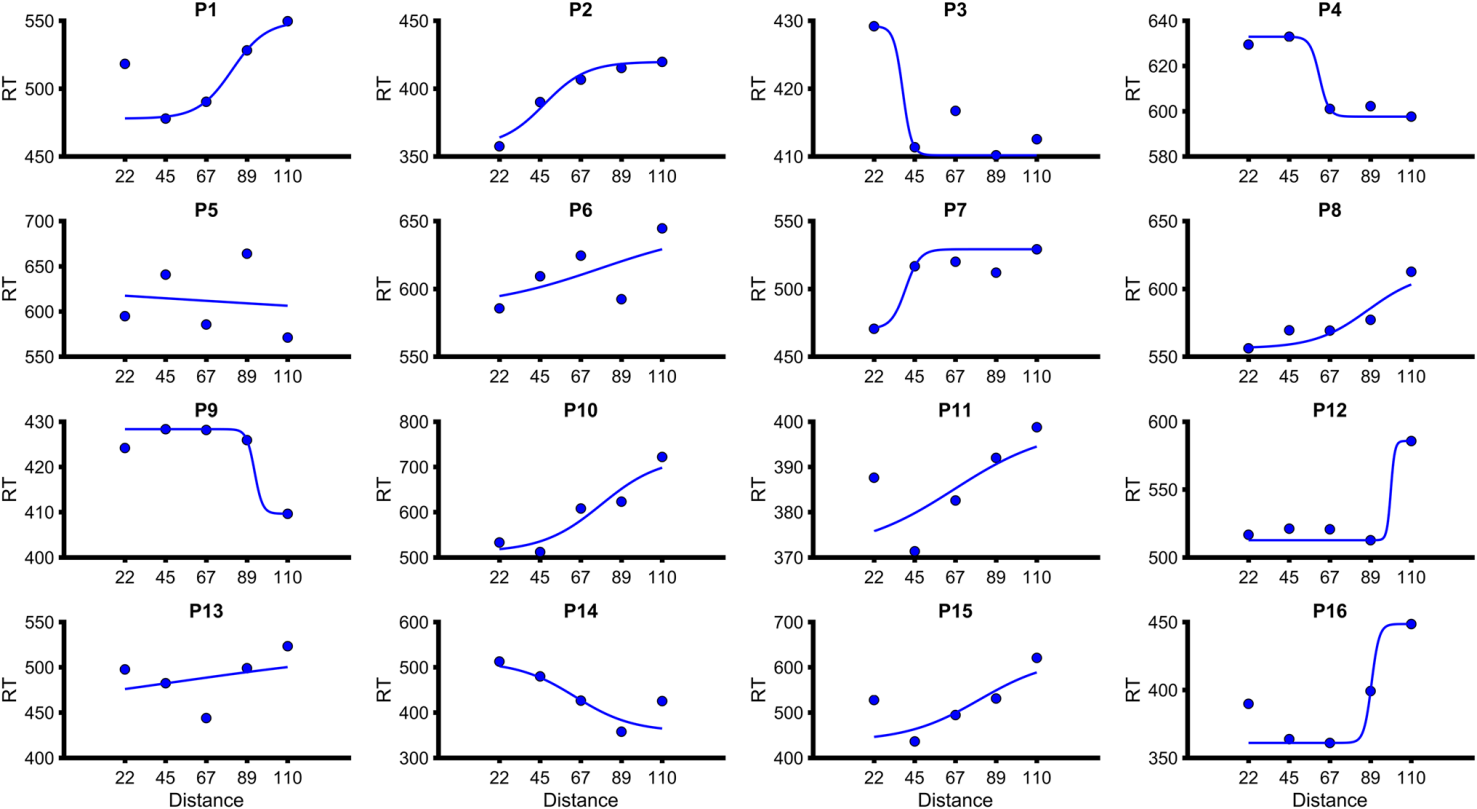
Mean reaction times (represented as blue opaque circles) and sigmoidal fits (blue lines) for the left leg of individual control participants (represented a *P* for participant followed by participant number). The *y*-axis represents the reaction time (RT) in milliseconds and the *x*-axis represents the distance from the foot (in centimeters) that the visual stimulus was located when the tactile stimulus was administered

*Left leg* Shapiro–Wilk tests revealed that *R*^2^ values for the sigmoidal fit were not normally distributed (*W* = 0.86, *p* = 0.025). Wilcoxon signed-rank tests revealed that *R*^2^ values for the sigmoidal fit (median = 0.80, IQR = 0.49–0.90) were significantly higher than for the linear (median = 0.45, IQR = 0.42–0.66) fit (*W* = 128, *p* < 0.001). Paired samples *t* tests revealed that RMSEs for the sigmoidal fits (19.5 ± 16.1 SD) were significantly lower than for the linear fits (24.0 ± 15.5 SD; *t*(15) = − 3.4, *p* = 0.004, *d* = − 0.8). These results suggest that the data were better explained by the sigmoidal function. Therefore, the central point of the sigmoid was taken as the PPS boundary (66.9 cm ± 22.6). See Fig. 3 for visualization of individual sigmoid fits.

*Right leg compared to left leg* A paired samples *t* test revealed no difference in PPS boundaries for the left and right legs (*t*(15) = − 0.3, *p* = 0.76, *d* = − 0.07). Sigmoid slope values were not normally distributed (*p* ≤ 0.02). Wilcoxon signed-rank tests revealed no difference in slope values (i.e. the ‘steepness’ of the change from PPS to extra-personal space) for the left (median = 0.9, IQR = − 0.4–2.0) and right (median = 0.6, IQR = 0.1–3.5) feet (*W* = 82, *p* = 0.4). Taken together, PPS around the left and right feet was similar for controls.

#### BIID participants

Mean reaction times per distance, per leg per participant (for bimodal visuo-tactile trials only, i.e. *D*1–*D*5 from Fig. 1) were fitted to linear and sigmoid fits for the BIID participants. Unlike controls, we did not conduct a formal statistical analysis comparing the two fits. To compare the BIID participants to controls, however, we took a pragmatic approach and used the sigmoid fit to explore the data further. RMSEs and *R*^2^ values for each BIID participant (as well as the mean and SD for controls) are shown in Table 3.

**Table 3:** Goodness-of-fit values for the right and left legs of BIID participants and controls (average and standard deviation)

Subjects	Right				Left			
	RMSE (ms)		*R*^2^		RMSE (ms)		*R*^2^	
	Sigmoid	Linear	Sigmoid	Linear	Sigmoid	Linear	Sigmoid	Linear
1-LA	22.4	22.2	− 0.01	0.00	8.3	11.4	0.80	0.62
2-LA	16.1	16.1	0.18	0.17	9.2	12.0	0.55	0.23
3-RA	30.1	35.6	0.77	0.67	8.2	15.8	0.93	0.75
Controls [ave. (sd)]	22.0 (13.3)	23.9 (12.0)	0.42 (0.37)	0.34 (0.31)	19.5 (16.1)	24.0 (15.5)	0.66 (0.30)	0.51 (0.24)

#### Comparisons to corresponding leg of control participants

Overall mean reaction times did not differ between each BIID participant and controls for the affected (*p* ≥ 0.1) nor for the unaffected (*p* ≥ 0.8) legs.

##### Curve fitting

**Fig. 4:**
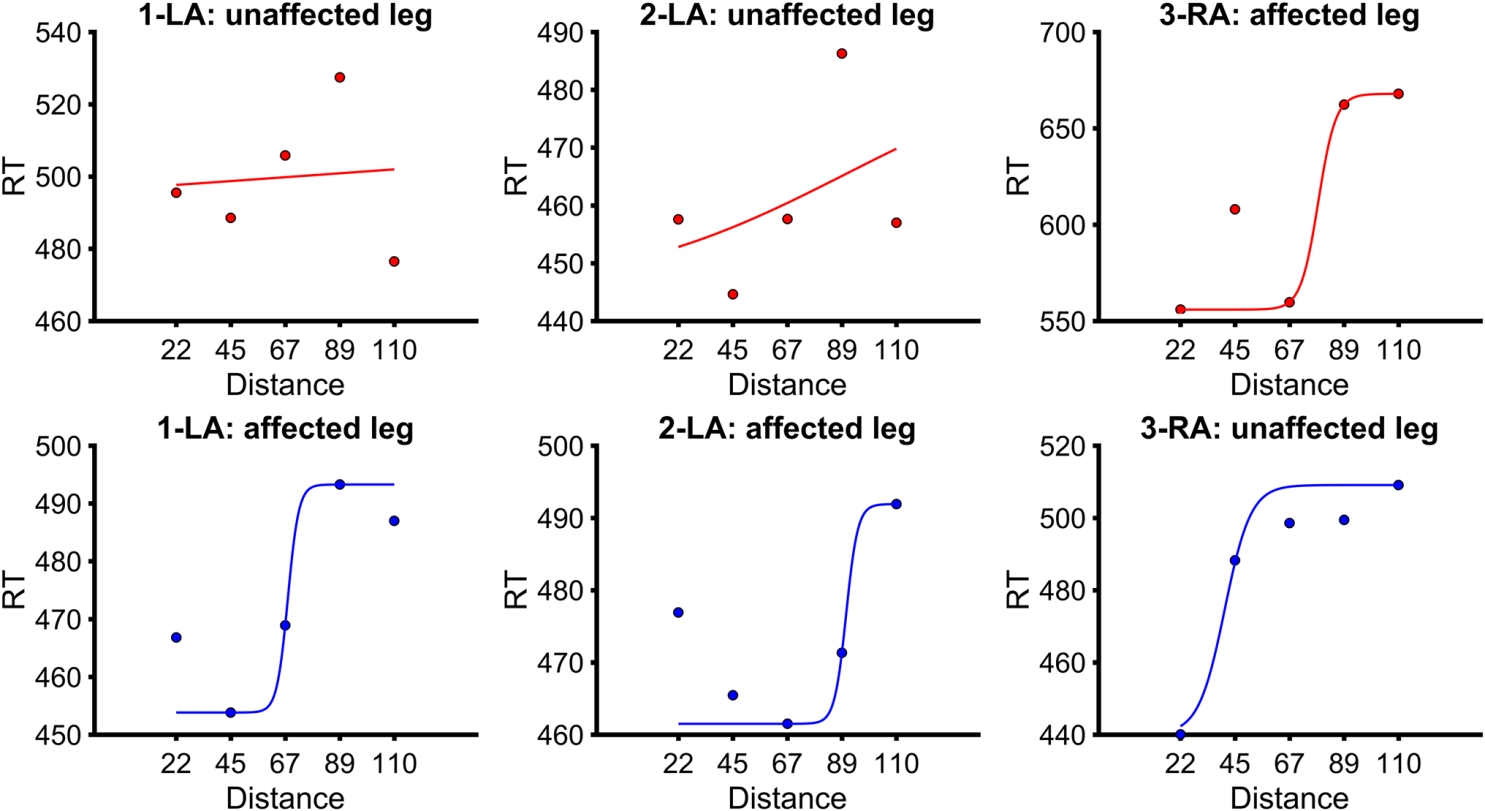
Mean reaction times (represented as opaque circles) and sigmoidal fits (for the right legs (top row, in red) and left legs (bottom row, in blue) of individual BIID participants (participant number and affected leg as title for each plot). The *y*-axis represents the reaction time (RT) in milliseconds and the *x*-axis represents the distance from the foot (in centimeters) that the visual stimulus was located when the tactile stimulus was administered

RMSEs, *R*^2^, slopes, and PPS boundaries (i.e. central point of the sigmoid(s)) for the left and right legs of each BIID participant were compared to the corresponding leg of controls. See Fig. 4 for mean reaction times per distance with individual sigmoid fits. One-tailed tests comparing the boundaries were conducted for the affected legs (as we hypothesized a smaller PPS for this leg) and two-tailed tests were conducted for the unaffected legs.

##### 1-LA

*Right leg (unaffected leg)* RMSE (22.3 ms) did not differ from controls (*p* = 0.84, effect size (*Z*-CC) = 0.02). *R*^2^ (− 0.08) did not differ from controls (*p* = 0.27, effect size (*Z*-CC) = − 1.16). However, since *R*^2^ was negative, we could not interpret the boundary or slope for this participant’s right leg. *Left leg (affected leg)* RMSE (8.3 ms) did not differ from controls (*p* = 0.50 effect size (*Z*-CC) = − 0.69). *R*^2^ (0.80) did not differ from controls (*p* = 0.67, effect size (*Z*-CC) = 0.41). The slope (9.97) also was not different from controls (*p* = 0.26, effect size (*Z*-CC) = 1.18). The PPS boundary (67.7 cm) was not significantly smaller than for controls (*p* = 0.42, effect size (*Z*-CC) = 0.03).

##### 2-LA

*Right leg (unaffected leg) *RMSE (16.1 ms) did not differ from controls (*p* = 0.66, effect size (*Z*-CC) = − 0.44). *R*^2^ (0.18) did not differ from controls (*p* = 0.53, effect size (*Z*-CC) = − 0.66). The slope (0.46) also was not different from controls (*p* = 0.55, effect size (*Z*-CC) = − 0.62). Finally, the PPS boundary (90.6 cm) was not different from controls (*p* = 0.43, effect size (*Z*-CC) = 0.83). *Left leg (affected leg) *RMSE (9.1 ms) did not differ from controls (*p* = 0.54, effect size (*Z*-CC) = − 0.64). *R*^2^ (0.55) did not differ from controls (*p* = 0.69, effect size (*Z*-CC) = − 0.38). The slope (9.7) also was not different from controls (*p* = 0.28, effect size (*Z*-CC) = 1.15). The PPS boundary (90.6 cm) was not significantly smaller than for controls (*p* = 0.16, effect size (*Z*-CC) = 1.04).

##### 3-RA

*Right leg (affected leg)* RMSE (30.0 ms) did not differ from controls (*p* = 0.56, effect size (*Z*-CC) = 0.60). *R*^2^ (0.77) did not differ from controls (*p* = 0.40, effect size (*Z*-CC) = 0.88). The slope (6.2) also was not different from controls (*p* = 0.31, effect size (*Z*-CC) = 0.50). The PPS boundary (78.4 cm) was not significantly smaller than for controls (*p* = 0.35, effect size (*Z*-CC) = 0.36). *Left leg (unaffected leg)* RMSE (8.1 ms) did not differ from controls (*p* = 0.49, effect size (*Z*-CC) = − 0.7). *R*^2^ (0.93) did not differ from controls (*p* = 0.41, effect size (*Z*-CC) = 0.86). The slope (4.1) also was not different from controls (*p* = 0.68, effect size (*Z*-CC) = 0.41). Finally, the PPS boundary (40.2 cm) was not different from controls (*p* = 0.26, effect size (*Z*-CC) = − 1.18).

#### Affected leg versus unaffected leg

The boundaries for the left and right legs for 2-LA and 3-RA were compared using Bayesian tests on the difference between the cases’ standardized scores (BSTD). Since 1-LA had a negative *R*^2^ for fitting both sigmoid and linear models, we could not compare the boundaries between his affected and unaffected leg.

##### 2-LA

*Left leg versus right leg PPS boundary* The difference between the case’s standardized scores was not statistically significant (one-tailed) on the BSTD (*p* = 0.42, effect size (*Z*-*D*CC) = − 0.21).

##### 3-RA

*Left leg versus right leg PPS boundary* The difference between the case’s standardized scores was not statistically significant (one-tailed) on the BSTD (*p* = 0.08, effect size (*Z*-*D*CC) = 1.515).

## Discussion

In the current study, we examined peripersonal space (PPS) around the lower limbs in three males with BIID, who desired unilateral amputation of one of their legs, and in 16 healthy male participants. We used a visuo-tactile interaction task to examine the average boundaries of the leg PPS. Participants were asked to respond to a vibration on their left or right toe while a task-irrelevant (animated) visual stimulus approached that same toe. The size of a person’s PPS can be extracted by examining the relationship between the proximity of the visual stimulus and the reaction times to the tactile stimuli. Recently, we found that reaction times to tactile stimuli around the lower limbs are faster when the visual stimulus is within about 75 cm from the toes (Stone, Kandula, Keizer, & Dijkerman, 2018b). We replicate these findings and also show that PPS size is similar for both legs. In addition, we expected to find a diminished PPS around the affected leg (i.e. the leg desired to-be-removed) in BIID participants compared to the corresponding leg of controls and their other (unaffected) leg. In contrast, we found that PPS around both affected and unaffected legs in (three men with) BIID did not differ from that of healthy controls. These findings extend our knowledge about lower limb PPS representations and provide insight into bodily self-consciousness in the rare condition of BIID.

We found that the size of PPS around the lower limbs in our healthy control group was around 70 cm (with a large standard deviation of ~ 25 cm). This finding is in line with our previous report (Stone, Kandula, Keizer, & Dijkerman, 2018b) wherein we looked at PPS around the legs as a whole, rather than for each leg separately. Moreover, this distance is similar to what has been found for PPS around the trunk (Noel et al., 2015a; Serino et al., 2016), which might share PPS with the lower limbs. Furthermore, we found that average PPS boundaries did not differ between the left (~ 67 cm) and right (~ 69 cm) legs nor did the average slopes (which reflects the overall shape of the PPS). This suggests that PPS, at least measured in a task such as this one, is similar for both legs. This is perhaps not surprising as actions made with the lower body are usually made in tandem and do not usually play different roles (except for, perhaps, during a sport). Although, asymmetries in left and right PPS have been revealed before. One study showed that right-handed people have a larger PPS on their left side, whereas left-handed people have a similar PPS for both sides of the body (Hobeika, Viaud-Delmon, & Taffou, 2018). Moreover, people who have had an upper limb amputation have a smaller PPS around the stump than for the intact limb (Canzoneri et al., 2013a). While studies investigating lower limb PPS around the amputated and intact limb are lacking, an unpublished case study from our lab revealed a normal PPS around the intact limb of a lower limb amputee. If PPS was one in the same for both legs (or arms in the aforementioned study), one might expect PPS for the intact limb to reduce in the absence of a limb. Taken together, we show that PPS is similar around each leg in healthy individuals.

We also investigated whether PPS around the legs was different in a small sample of men with BIID. People with BIID, particularly those who desire amputation of a limb, feel like that limb is foreign and does not belong do them, stating that they are ‘overcomplete’ with the limb (e.g. Blom, Hennekam, & Denys, 2012). However, this disturbed feeling of ownership over the body part is not delusional—they know that the part is physically attached to them, functions fine, and is under their control (Brugger, Christen, Jellestad, & Hänggi, 2016). It remains unknown why BIID manifests itself. However, many studies have suggested that it could be due to a (probably congenital) disturbed representation of the body (part) in the brain (Blom et al., 2016b; Hänggi, Bellwald, & Brugger, 2016; Hänggi et al., 2017; Hilti et al., 2013; McGeoch et al., 2011; Oddo-Sommerfeld et al., 2018; van Dijk et al., 2013). This produces a mismatch between how the body physically is and how the individual internally perceives it should be. This results therefore in a desire to abolish the structure and/or function of that part so as to be aligned with the internal representation. What consequences might ensue from this disturbed internal representation? Interestingly, two studies have revealed that the physiological response to stimuli approaching and contacting the affected leg in people with a unilateral lower limb amputation desire is different than for the unaffected leg and the corresponding leg of healthy controls (Brang, McGeoch, & Ramachandran, 2008; Romano, Sedda, Brugger, & Bottini, 2015). That is, approaching (but not contacting) the unwanted (affected) leg showed a reduced skin conductance response (SCR) in comparison to the unaffected leg (Romano, Sedda, Brugger, & Bottini, 2015). What is more, contacting the unwanted leg elicited an exaggerated SCR (compared to the unaffected leg), as if the brain did not predict the touch, even though the approach of the stimulus (pin and cotton swab) was in full view. This failure to ‘predict touch’ on the affected part suggests that PPS around that affected part might be different in individuals with BIID. Specifically, if there is a reduced SCR to stimuli approaching the affected limb, then perhaps PPS is diminished around the part. Analysis of the size of PPS around each BIID limb did not show differences in comparison to controls or to the other leg (at least in 2-LA and 3-RA, as 1-LA’s data were not suitable to fit a sigmoidal curve, see below for discussion). PPS seemed to be overall ‘normal’ for the BIID leg, at least using this type of measurement, suggesting that multisensory integration of stimuli still occurs at a faster rate near the leg compared to farther away, regardless of whether or not the individual desires amputation of that leg.

Our data were seemingly in conflict with these previous studies. In our study, participants were asked to press a button whenever they felt a tactile stimulus on their toes. We used neutral tactile and visual stimuli in our study, while Romano et al. used a neutral (cotton swab) and a noxious (pin) stimulus. While their results did not show an interaction between stimulus type and side (affected leg or unaffected leg), they did show an interaction between stimulus type and whether or not the stimulus contacted (or simply approached) the limb (i.e. contact type). Specifically, noxious stimuli elicited stronger SCR responses to touching (*z* score of SCR: 0.66) than to simply approaching (*z* score of SCR: 0.06) the limb (regardless of limb), whereas neutral stimuli did not elicit discrepant SCRs between touching (*z* score of SCR: − 0.34) and simply approaching (*z* score of SCR: − 0.37) the legs. Their results, however, are discussed in terms of the interaction between contact type (touch or approach) and side (affected or unaffected) leg, so the results are collapsed across stimulus type. Recently, Bufacchi and Iannetti (2018) argued that PPS size depends not only on the proximity of a stimulus but also on its behavioral relevance to a given action or set of actions. The stimuli in our study were behaviourally relevant to prepare button presses made with the hand, but not for preparing to retract the legs from something threatening, per se. Thus, an investigation of PPS in BIID with threatening (noxious) stimuli instead therefore might provide results more in line with a diminished PPS around the unwanted leg. Moreover, it is possible that our behavioral outcome of PPS (in terms of reaction times) is not sensitive enough to capture differences in PPS around the affected body part in BIID. Future studies should include SCR as an additional measure to address this possibility.

But why would the brain still maintain some form of PPS around a leg that presumably does not belong to the body (or is not properly inscribed into the body representation)? We provide three non-mutually exclusive explanations for this. First, it is possible that the brain treats the leg as a sensorimotor tool. Several behavioral studies have shown that PPS extends when people use a tool, such as a computer mouse (Bassolino, Serino, Ubaldi, & Ladavas, 2010), wheelchair (Galli et al., 2015), a long stick (Canzoneri et al., 2013b), or prosthetic limb (Canzoneri et al., 2013a). For instance, use of a 1-m stick to retrieve target objects for 20 min led to an increase of PPS around the hand, suggesting that PPS changed to incorporate the tool into its representation. Moreover, the size of the stunted PPS around the amputated upper limb ‘expanded’, to some extent, when upper-limb amputees wore a prosthetic hand (Canzoneri et al., 2013a). At the neurophysiological level, visual PPS neurons anchored to the hand in non-human primates elongate after use of a tool to retrieve food (Iriki, Tanaka, & Iwamura, 1996). So perhaps in the case of BIID, because the leg is physically present and processes primary sensory feedback normally, it repurposes itself as a tool, thereby resulting in normal PPS around the leg (even in the absence of ownership over that leg). Since the leg still maintains the possibility to act, revealed by the fact that the individual can still walk and use the limb, this might be sufficient to uphold a PPS representation. For example, passively moving the legs of paralyzed individuals restores PPS around the legs (Scandola et al., 2016), emphasizing the tight link between PPS and action. A second explanation as to why a PPS might be maintained in the absence of ownership is the physical and sensory congruence of the leg with the body. Individuals with BIID who desire amputation of a limb often state that the limb feels foreign to their bodies. In line with this, it has been shown that placing a fake, thereby foreign, arm within the space of the real arm of a monkey elicits multisensory responses in about a quarter of the PPS neurons, suggesting that the visuo-proprioceptive congruence is sufficient to incorporate a limb into PPS (Graziano, Yap, & Gross, 1999; Graziano, Cooke, & Taylor, 2000). So, while the limb might feel like it does not belong to the body in BIID, the peripersonal space network, through its physical appearance and congruent multisensory input might still process the limb as part of oneself. In turn, this facilitates visuo-tactile integration near that part. Moreover, a recent meta-analysis showed that the brain areas responsible for feeling of body ownership and peripersonal space are significantly dissociated for the most part (Grivaz, Blanke, & Serino, 2017). People with amputation variant BIID often report feelings of disturbed body ownership over the limb, so it is possible that the brain areas implicated in BIID are also significantly dissociated from PPS, at least at a functional level. Finally, the leg PPS could be merging with the trunk PPS. When the hand is placed in front of the trunk, it merges with the trunk space (Serino et al., 2016). Similarly, when the hand is placed in front of the face, the hand blink reflex (another common measure of PPS) increases (Sambo, Liang, Cruccu, & Iannetti, 2012). Perhaps in this case, the trunk PPS ‘takes care’ of the leg PPS in BIID. Due to anatomical constraints, it is difficult to displace one leg’s position laterally for a long period of time, making this hypothesis challenging to test. Alternatively, though, it is possible that PPS is simply unimpaired in BIID.

A brief discussion of PPS around the unaffected (normal) leg in our sample of BIID participants is also warranted. In fact, visual inspection of Fig. 4 suggests that the sigmoidal fits for the affected leg appear to be better than for the unaffected leg, contrary to what we expected. One study showed that people with BIID have a more pronounced rubber foot illusion for the foot that corresponds to their affected side (Lenggenhager, Hilti, & Brugger, 2015). In this illusion, participants view a rubber foot being synchronously stroked with their own, unseen, real foot. This conflicting visual and tactile information is reconciled by referring the felt touch to where the touch is seen, leading to a feeling of ownership over the fake foot. Importantly, this illusion involves the integration of visual input of the fake foot with tactile signals on the real foot within the PPS of the body part (Preston 2013). Perhaps visuo-tactile integration around the unaffected leg is less distinct/pronounced for PPS processing (or that attentional mechanisms facilitate visuo-tactile integration for the affected part, e.g. see Aoyama et al., 2012). Moreover, when the visual stimulus was located at its farthest point from the toes, tactile reaction times for the unaffected legs of 1-LA and 2-LA were particularly fast (resulting in disrupted curve fits). One can envision that if these response times mimicked the next closest distance (89 cm), then a PPS pattern would be clear here. The reasons for these quick response times at the start of the trial are unknown, but this pattern of responses was not unlike some of the control participants (e.g. right foot of P-8, P-15, or left foot of P-5, see Figs. 2, 3). It is possible that external factors, such as those related to sustained attention, in our participants might have influenced these reaction times. However, we cannot be certain as we did not measure or account for other variables (e.g. caffeine intake prior to testing, levels of anxiety, arousal level, etc.).

As BIID is such a rare (and secretive) condition, recruitment of sample sizes of homogenous types of BIID sufficient for a group-level analysis is a challenge. Several other studies investigating BIID have faced similar challenges (e.g. Aoyama et al., 2012, *n* = 5; Brang, McGeoch, & Ramachandran, 2008, *n* = 2; Bottini, Brugger, & Sedda, 2015, *n* = 7; van Dijk et al., 2013, *n* = 5) with many being case studies (Bensler & Paauw, 2003; Braam, Visser, Cath, & Hoogendijk, 2005; Everaerd, 1983; Parsons, Brown, & Sirota, 1981; Storm & Weiss, 2003). While three individuals might not be representative of the entire BIID population, their patterns of behavior are overall similar, particularly for the affected leg. This incites some level of confidence in concluding that PPS is ‘normal’ for the BIID leg. However, this task might be more suitable to use at a group level, rather than at an individual level. Visualization of the individual fits (Figs. 2, 3) of the control sample show large variability in response patterns at the individual level, even between one’s own legs. Studies have shown that several factors can influence the size of one’s PPS, for example, individual differences in brain activity (Ferri et al., 2015a), emotional states such as anxiety (Sambo & Iannetti, 2013) or fear (Ferri et al., 2015b; de Haan, Smit, van der Stigchel, & Dijkerman, 2016; Taffou & Viaud-Delmon, 2014), one’s level of interoceptive accuracy (Ardizzi & Ferri, 2018), anxiety disorders such as claustrophobia (Hunley, Marker, & Lourenco, 2017). However, the desire to amputate a healthy leg does not seem to be one of these factors, at least with use of this current measure.

In conclusion, the size of PPS around the left and right legs is similar in healthy individuals. Moreover, we found that the size (and shape) of PPS around the unwanted leg in BIID did not differ from that of controls and of the unaffected leg. This implies that visuo-tactile processing of neutral stimuli around the leg is normal in BIID. So, while the limb might feel foreign to the individual, the brain still seems to integrate multisensory input near that leg. These results might reflect and reiterate the feeling of ‘overcompleteness’ that people with BIID experience—such that sensory information about the leg is still processed to act with and protect the leg, but the internal experienced representation is that of a congenital amputee. As one of our BIID participants in the current reported stated: “In my head it feels like my right leg is amputated above the knee”. Future research uncovering the foundation of such statements is needed to understand the mechanisms that drive this condition. Specifically, this research should focus on correlating physiological responses (through SCR or neuroimaging techniques, for example) with one’s subjective perception of their BIID.

## References

[CR1] Aoyama A, Krummenacher P, Palla A (2012). Impaired spatial-temporal integration of touch in xenomelia (body integrity identity disorder). Spatial Cognition & Computation.

[CR2] Ardizzi M, Ferri F (2018). Interoceptive influences on peripersonal space boundary. Cognition.

[CR3] Bassolino M, Serino A, Ubaldi S, Làdavas E (2010). Everyday use of the computer mouse extends peripersonal space representation. Neuropsychologia.

[CR4] Bensler JM, Paauw DS (2003). Apotemnophilia masquerading as medical morbidity. (Case report). Southern Medical Journal.

[CR5] Blom RM, Guglielmi V, Denys D (2016). Elective amputation of a “healthy limb”. CNS Spectrums.

[CR6] Blom RM, Hennekam RC, Denys D (2012). Body integrity identity disorder. PLoS ONE.

[CR7] Blom RM, van der Wal SJ, Vulink NC, Denys D (2017). Role of sexuality in body integrity identity disorder (BIID): A cross-sectional internet-based survey study. The Journal of Sexual Medicine.

[CR8] Blom RM, Van Wingen GA, Van Der Wal SJ (2016). The desire for amputation or paralyzation: Evidence for structural brain anomalies in body integrity identity disorder (BIID). PLoS ONE.

[CR9] Bottini G, Brugger P, Sedda A (2015). Is the desire for amputation related to disturbed emotion processing? A multiple case study analysis in BIID. Neurocase.

[CR10] Braam AW, Visser S, Cath DC, Hoogendijk WJG (2005). Investigation of the syndrome of apotemnophilia and course of a cognitive-behavioural therapy. Psychopathology.

[CR11] Brang D, McGeoch PD, Ramachandran VS (2008). Apotemnophilia: A neurological disorder. NeuroReport.

[CR12] Bremmer F, Schlack A, Shah NJ (2001). Polymodal motion processing in posterior parietal and premotor cortex: A human fMRI study strongly implies equivalencies between humans and monkeys. Neuron.

[CR13] Brozzoli C, Makin TR, Cardinali L, Murray MM, Wallace MT (2012). Peripersonal space: A multisensory interface for body–object interactions. The neural bases of multisensory processes.

[CR14] Brugger P, Christen M, Jellestad L, Hänggi J (2016). Limb amputation and other disability desires as a medical condition. The Lancet Psychiatry.

[CR15] Brugger P, Lenggenhager B, Giummarra MJ (2013). Xenomelia: A social neuroscience view of altered bodily self-consciousness. Front Psychol.

[CR16] Bufacchi RJ, Iannetti GD (2018). An action field theory of peripersonal space. Trends in Cognitive Sciences.

[CR17] Canzoneri E, Magosso E, Serino A (2012). Dynamic sounds capture the boundaries of peripersonal space representation in humans. PLoS ONE.

[CR18] Canzoneri E, Marzolla M, Amoresano A (2013). Amputation and prosthesis implantation shape body and peripersonal space representations. Scientific Reports.

[CR19] Canzoneri E, Ubaldi S, Rastelli V (2013). Tool-use reshapes the boundaries of body and peripersonal space representations. Experimental Brain Research.

[CR20] Crawford JR, Garthwaite PH (2007). Comparison of a single case to a control or normative sample in neuropsychology: Development of a Bayesian approach. Cognitive Neuropsychology.

[CR21] Crawford JR, Garthwaite PH, Porter S (2010). Point and interval estimates of effect sizes for the case-controls design in neuropsychology: Rationale, methods, implementations, and proposed reporting standards. Cognitive Neuropsychology.

[CR22] de Haan AM, De SM, van der Stigchel S, Dijkerman HC (2016). Approaching threat modulates visuotactile interactions in peripersonal space. Experimental Brain Research.

[CR23] Everaerd W (1983). A case of apotemnophilia: A handicap as sexual preference. American Journal of Psychotherapy.

[CR24] Ferri F, Costantini M, Huang Z (2015). Intertrial variability in the premotor cortex accounts for individual differences in peripersonal space. Journal of Neuroscience.

[CR25] Ferri F, Tajadura-Jiménez A, Väljamäe A (2015). Emotion-inducing approaching sounds shape the boundaries of multisensory peripersonal space. Neuropsychologia.

[CR26] First, M. B. (2015). Structured clinical interview for the DSM (SCID). *The Encyclopedia of Clinical Psychology *(pp. 1–6).

[CR27] First MB, Fisher C (2012). Body integrity identity disorder: The persistent desire to acquire a physical disability. Psychopathology.

[CR28] Fogassi L, Gallese V, Fadiga L (1996). Coding of peripersonal space in inferior premotor cortex (area F4). Journal of Neurophysiology.

[CR29] Galli G, Noel J-P, Canzoneri E (2015). The wheelchair as a full-body tool extending the peripersonal space. Frontiers in Psychology.

[CR30] Giummarra MJ, Bradshaw JL, Hilti LM (2012). Paralyzed by desire: A new type of body integrity identity disorder. Cognitive Behavioral Neurology.

[CR31] Graziano MS (1999). Where is my arm? The relative role of vision and proprioception in the neuronal representation of limb position. Proceedings of the National Academy of Sciences of the United States of America.

[CR32] Graziano MS, Cooke DF, Taylor CS (2000). Coding the location of the arm by sight. Science.

[CR33] Graziano MS, Hu XT, Gross CG (1997). Visuospatial properties of ventral premotor cortex. Journal of Neurophysiology.

[CR34] Graziano MS, Yap GS, Gross CG (1994). Coding of visual space by premotor neurons. Science.

[CR35] Grivaz P, Blanke O, Serino A (2017). Common and distinct brain regions processing multisensory bodily signals for peripersonal space and body ownership. Neuroimage.

[CR36] Hänggi J, Bellwald D, Brugger P (2016). Shape alterations of basal ganglia and thalamus in xenomelia. NeuroImage: Clinical.

[CR37] Hänggi J, Vitacco DA, Hilti LM (2017). Structural and functional hyperconnectivity within the sensorimotor system in xenomelia. Brain and Behavior.

[CR38] Hilti LM, Hänggi J, Vitacco DA (2013). The desire for healthy limb amputation: Structural brain correlates and clinical features of xenomelia. Brain.

[CR39] Hobeika L, Viaud-Delmon I, Taffou M (2018). Anisotropy of lateral peripersonal space is linked to handedness. Experimental Brain Research.

[CR40] Huang R-S, Chen C, Tran AT (2012). Mapping multisensory parietal face and body areas in humans. Proceedings of the National Academy of Sciences of the United States of America.

[CR41] Hunley SB, Marker AM, Lourenco SF (2017). Individual differences in the flexibility of peripersonal space. Experimental Psychology.

[CR42] Iriki A, Tanaka M, Iwamura Y (1996). Coding of modified body schema during tool use by macaque postcentral neurones. NeuroReport.

[CR43] Kandula M, Hofman D, Dijkerman HC (2015). Visuo-tactile interactions are dependent on the predictive value of the visual stimulus. Neuropsychologia.

[CR44] Kandula M, van der Stoep N, Hofman D, Dijkerman HC (2017). On the contribution of overt tactile expectations to visuo-tactile interactions within the peripersonal space. Experimental Brain Research.

[CR45] Làdavas E, di Pellegrino G, Farnè A, Zeloni G (1998). Neuropsychological evidence of an integrated visuotactile representation of peripersonal space in humans. Journal of Cognitive Neuroscience.

[CR46] Làdavas E, Pavani F, Farnè A (2001). Auditory peripersonal space in humans: A case of auditory–tactile extinction. Neurocase.

[CR47] Lenggenhager B, Hilti LM, Brugger P (2015). Disturbed body integrity and the “rubber foot illusion”. Neuropsychology.

[CR48] Makowski D (2018). The psycho package: An efficient and publishing-oriented workflow for psychological science. Journal of Open Source Software.

[CR49] McGeoch PD, Brang D, Song T (2011). Xenomelia: A new right parietal lobe syndrome. Journal of Neurology, Neurosurgery and Psychiatry.

[CR50] Noel J-P, Grivaz P, Marmaroli P (2015). Full body action remapping of peripersonal space: The case of walking. Neuropsychologia.

[CR51] Noel J-P, Pfeiffer C, Blanke O, Serino A (2015). Peripersonal space as the space of the bodily self. Cognition.

[CR52] Noll S, Kasten E (2014). Body integrity identity disorder (BIID): How satisfied are successful wannabes. Psychology and Behavioral Sciences.

[CR53] Oddo-Sommerfeld S, Hänggi J, Coletta L (2018). Brain activity elicited by viewing pictures of the own virtually amputated body predicts xenomelia. Neuropsychologia.

[CR54] Parsons JA, Brown WA, Sirota AD (1981). Inappropriate amputation requests. Psychosomatics.

[CR55] Preston C (2013). The role of distance from the body and distance from the real hand in ownership and disownership during the rubber hand illusion. Acta Psychologica.

[CR56] Ramachandran VS, Brang D, McGeoch PD, Rosar W (2009). Sexual and food preference in apotemnophilia and anorexia: Interactions between “beliefs” and “needs” regulated by two-way connections between body image and limbic structures. Perception.

[CR57] Rizzolatti G, Scandolara C, Matelli M, Gentilucci M (1981). Afferent properties of periarcuate neurons in macaque monkeys I. Somatosensory responses. Behavioural Brain Research.

[CR58] Romano D, Sedda A, Brugger P, Bottini G (2015). Body ownership: When feeling and knowing diverge. Consciousness and Cognition.

[CR59] Salomon R, Noel J-P, Łukowska M (2017). Unconscious integration of multisensory bodily inputs in the peripersonal space shapes bodily self-consciousness. Cognition.

[CR60] Sambo CF, Iannetti GD (2013). Better safe than sorry? The safety margin surrounding the body is increased by anxiety. Journal of Neuroscience.

[CR61] Sambo CF, Liang M, Cruccu G, Iannetti G (2012). Defensive peripersonal space: The blink reflex evoked by hand stimulation is increased when the hand is near the face. Journal of Neurophysiology.

[CR62] Scandola M, Aglioti SM, Bonente C (2016). Spinal cord lesions shrink peripersonal space around the feet, passive mobilization of paraplegic limbs restores it. Scientific Reports.

[CR63] Serino A, Noel J-P, Galli G (2016). Body part-centered and full body-centered peripersonal space representations. Scientific Reports.

[CR64] Sheehan DV, Lecrubier Y, Sheehan KH (1998). The mini-international neuropsychiatric interview (M.I.N.I.): The development and validation of a structured diagnostic psychiatric interview for DSM-IV and ICD-10. Journal of Clinical Psychiatry.

[CR65] Stone KD, Bullock F, Keizer A, Dijkerman HC (2018). The disappearing limb trick and the role of sensory suggestibility in illusion experience. Neuropsychologia.

[CR66] Stone KD, Kandula M, Keizer A, Dijkerman HC (2018). Peripersonal space boundaries around the lower limbs. Experimental Brain Research.

[CR67] Storm S, Weiss MD (2003). Self-inflicted tourniquet paralysis mimicking acute demyelinating polyneuropathy. Muscle and Nerve.

[CR68] Taffou M, Viaud-Delmon I (2014). Cynophobic fear adaptively extends peri-personal space. Frontiers in Psychiatry.

[CR69] van Dijk MT, van Wingen GA, van Lammeren A (2013). Neural basis of limb ownership in individuals with body integrity identity disorder. PLoS ONE.

